# Ablation of Complex Fractionated Atrial Electrograms in Catheter Ablation for AF; Where have we been and where are we going?

**DOI:** 10.2174/157340312803760848

**Published:** 2012-11

**Authors:** Jane Caldwell, Damian Redfearn

**Affiliations:** Arrhythmia Service, Division of Cardiology, Kingston General Hospital, Queen’s University, 76 Stuart St, Kingston, Ontario, Canada

**Keywords:** Atrial fibrillation, complex fractionation, catheter ablation.

## Abstract

Catheter ablation for persistent AF remains a challenge to the ablator as the disease is now outside the veins and cannot be tackled by pulmonary vein isolation alone. In this article we describe targeting complex fractionated atrial electrograms (CFAE) as a method to guide atrial substrate modification.

## INTRODUCTION

**Fig. (1):**
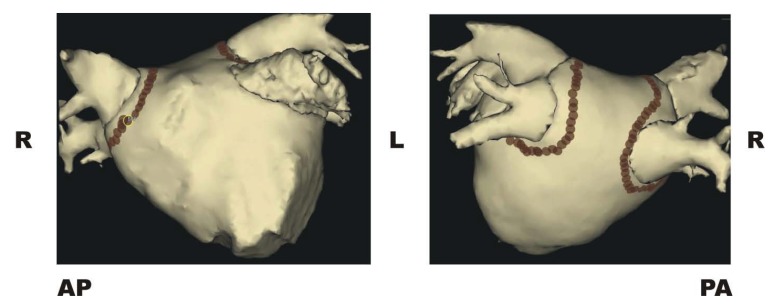
This virtual image of the left atrial endocardial surface was created by EnSite (St Jude’s Medical, St Paul’s, MN, USA). The image on the left is antero-posterior projection (**AP**) and on the right is postero-anterior (**PA**). The brown spots represent lesions made using radiofrequency application to circumferentially isolate the right (**R**) and left (**L**) pulmonary veins.

In the western world, atrial fibrillation (AF) is the most common sustained arrhythmia affecting 1-2% of the general population with a prevalence that is steadily increasing with the ever-ageing population [1]. Contemporary management of paroxysmal AF involves catheter ablation for symptomatic patients as the long-tem efficacy of antiarrhythmic therapy is disappointing with at best 40% of patients remaining responsive to antiarrhythmic drugs at 1 year [1,2]. The main target of AF catheter ablation is to electrically isolate the pulmonary veins, which has been most readily and reliably achievable by delivering radiofrequency ablation to electrically isolate the pairs of pulmonary veins from the remainder of the atrium i.e. circumferential pulmonary vein antral isolation (cPVAI) as shown in (Fig. **1**) [3]. Whilst the success rates for procedures treating paroxysmal AF are encouragingly high (67-89% free of AF at 1 year [1,2]), the outcomes for persistent AF procedures still lag well behind with freedom from AF after a single procedure varying between 21-56% (for systematic review see [4]). By the time AF has become persistent the disease process has greatly altered the atrial tissue itself via electrical and structural remodelling [5]. In trying to tackle this substrate remodelling ablators have targeted areas other than the pulmonary veins. One of these targets has been complex fractionated atrial electrograms (CFAEs). This review will look at how the idea of tracking and ablating CFAE arose, where we are currently at in terms of our understanding of CFAE and what they represent, and a look toward future directions.

## THE BEGINNINGS

**Fig. (2):**
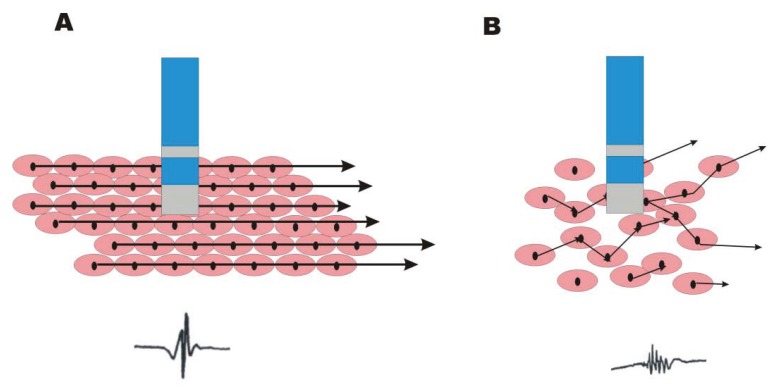
Panel **A** represents normal myocardium with fast synchronised activation of well connected mycocytes. The underlying bipolar electrogram is sharp and discrete. Panel **B** represents diseased myocardium where the intercellular connectivity is poor. The now dyssynchronous myocyte activity gives low amplitude, fractionated bipolar signals.

The term fractionation describes any process used to separate an entity into its constituent parts e.g. the fractionation of crude oil. In cardiac electrophysiology, whilst the initial term was used to describe the appearance of bipolar electrograms observed in animal models of ischaemia [6], perhaps the phenomenon would be better thought of as separation of the co-ordinated cardiac contraction into its individual myocyte depolarization by poor cell-to-cell connection. Thus there is no longer a unified depolarisation but rather ‘stuttering’ electrical propagation between myocytes (Fig. **2**). Although such fragmentation was originally described in animal model studies investigating arrhythmogenesis during myocardial infarction [6], the terminology was applied to signals recorded during open heart surgery in humans with AF [7].

Over the years, the definition and detection of complex fractionated atrial electrograms (CFAEs) has drastically changed. Originally, fractionated signals were manually detected and assigned with each study group having their own unique definition. For example, Jais *et al. *defined CFAE as “*continuous electrical activity or FF interval <100ms*” with recording for 60s with FF interval being the AF cycle length [8], whilst Nadamanee defined them as electrograms composed of ≥2 deflections, perturbation of the baseline with continuous deflection of a prolonged activation complex, or atrial electrograms with a cycle length ≤120 ms in low-voltage areas (i.e. <0.15 mV) in 10s recordings [9]. Alternative definitions were “*fractionated potentials exhibiting multiple deflections from the isoelectric line (≥3 deflections) and/or potentials with continuous electrical activity without an isoelectric line*” by Rostock *et al. *[10] or “*a cycle length≤120 ms or shorter than the coronary sinus, or those that were fractionated or displayed continuous electrical activity*” detected over an unspecified time period by Oral *et al. *[11]. 

**Fig. (3):**
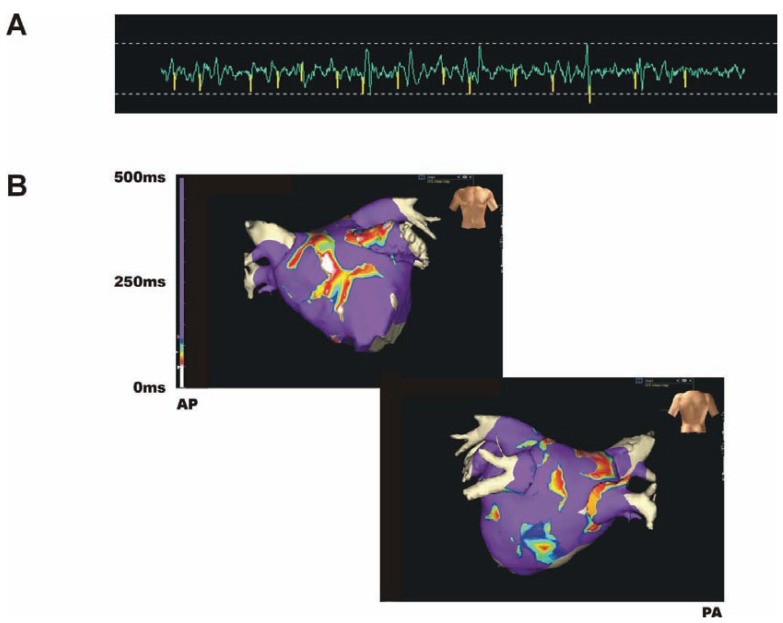
CFAE map generated by automatic detection of CFAE mean ≤120ms and projected onto the virtual endocarcial 3D surface of the left atrium by EnSite (St Jude’s Medical, St Paul’s, MN, USA). Panel **A** shows a typical bipolar endocardial signal with the yellow spikes where CFAE has been detected. Panel **B** shows the colour maps created by the software. The left upper image is antero-posterior projection (**AP**) whilst the right lower is postero-anterior (**PA**). The purple areas are non-CFAE where the mean interval is greater than 120ms. The colour scale for the CFAE runs from the shortest (white) to the longest (blue) as shown by the rainbow scale on the edge of the AP image.

Such variable definitions were not helpful in the early stages of fractionation research. Thus, in an attempt to unify detection, industry partners developed automated detection algorithms and incorporated them into the electroanatomical mapping systems employed in catheter ablation of atrial fibrillation. The 2 most commonly used mapping systems are EnSite (St Jude’s Medical, St Paul’s, MN, US) and CARTO (Biosense Webster, Diamond Bar, CA, US). With EnSite, fractionation is determined by the average interval between deflections, the ‘CFE mean diagnostic landmark map’, with CFAE generally considered to occur when the mean is ≤120ms over a 5 second epoch [12]. Whilst the analysis time interval can be altered by the operator up to 8s, 5-6s intervals are the most widespread used as they have been shown to be the most reproducible over time [12]. The software also allows determination of standard deviation of the average deflection interval and calculation of the peak or dominant frequency of the complex signal but these are presently used only as research tools. In the CARTO system, fractionation is generally determined by the shortest complex interval (SCI) between two consecutive deflections over a measuring period of ~2.5s [13]. The CARTO software can also measure CFAE by the (i) Average Complex Interval (ACI), which measures the average value for all the intervals between consecutive deflection within the time period, or the (ii) Interval Confidence Level (ICL) that measures the total number of intervals at each point in the given time period [14]. Both EnSite and CARTO display the results as a colour scale map on their virtual endocardial geometry (Fig. **3**).

Whilst the creation of automatic detection has arguably helped to reduce intra and interobserver variability, it has in no way removed it, as the assignation cut-off and time windows used vary between users. The degree of fractionation detected by a catheter will also vary depending on the separation between the poles on the catheter and whether mapping is performed bipolar (most common) or unipolar (as per Konings *et al. *original study [15]). Another consideration is that ablation itself alters the distribution of CFAE as recently highlighted by Matsuo *et al*. [16] who demonstrated the reduction in CFAE on remapping post PVI and post linear roof line ablation. For this reason ablators will often quickly “eyeball” the CFAE area before ablating and use the map as a guide. Similarly if they come across highly fractionated areas not indicated in the CFAE map many ablators will consider ablating these areas as well. So in summary, there is variable adoption of these automated tools with many manuscripts continuing to employ operator defined methodology [17-19]. 

Such variability was, and continues to be problematic in CFAE research. Another area of diversity is the nature of the causal substrate that underlies the CFAE; different well-respected groups have provided elegant evidence for their own hypothesis on the aetiology of CFAE. One of the first formative mechanistic studies was performed on humans at open heart surgery for accessory bypass tract ablation [15]. Using a spoon-shaped array unipolar of 244 electrodes Konings *et al. *defined the recorded electrograms during induced AF as either single potential, double potential (short or long) or fractionated potential. By forming isochronal maps they discovered that the fractionated potentials were observed where multiple wavelets were travelling through areas of slow conduction. Such observations fit with the hypothesis that AF is driven and maintained by multiple wavelets (for review of mechanisms of AF see Jalife *et al*. 2002 [20]) and that areas that generate these wavelets are key targets for ablation. In a slightly different vane, Kalife *et al. *found in isolated sheep atria that fractionation was highest at boundaries of dominant frequency domains [21]. Domains of dominant frequency form because of differences in electrophysiological properties of adjacent myocardium i.e. different refractoriness or different conduction velocities. When the activation front crosses from one boundary into another, there will be interruption of the wavefront resulting in fractionation. These observations more fit with the concept of AF being driven by mother rotors which then fractionate as the wavefront hits differing regions of refractoriness or conduction velocity [20]. This concept would suggest that fractionation is more a bystander phenomenon and thus not a good target for ablation. A third proposed underlying mechanism of AF is the requirement of autonomic stimulation to drive conversion of pulmonary focal triggers into widespread chaotic atrial activity [22]. This group also propose that CFAE are associated with the underlying ganglia [23]. Whilst the common CFAE sites are located near to parasympathetic ganglia [24] (Table **1**) and ablation of ganglia can terminate AF in canine models [23], this theory does not explain why CFAE are more widespread in persistent than paroxysmal AF [9]. In reality CFAEs are probably due to all 3 of these mechanisms with different contributions in different clinical scenarios. For example, it is likely that the majority of CFAEs in paroxysmal AF are bystanders resulting from “normal” atrial electrical heterogeneity in healthy high-voltage, unscarred tissue, manifesting at very short cycle lengths. This might also explain why catheter ablation of CFAE in paroxysmal AF has not been linked to improved AF free outcomes [25]. In patients with vagal paroxysmal AF, one could speculate that the parasympathetic ganglia play a more dominant role in CFAE formation. Indeed, in patients with vagal paroxysmal AF targeting the ganglia, rather than the CFAE per say, has been effective in aborting AF [26]. The difficult scenario is in persistent AF, where CFAE are more bountiful [27,28] and are likely a mix of bystander, healthy tissue and causal (and probably fibrosed [29]) tissue. 

The highly variable CFAE definitions and origins partly explain the inconsistent clinical experience of targeting CFAE for ablation and the differing theoretical explanations for the observed complex signals. The first study to target CFAE alone did so by manual identification of CFAE and achieved outstanding results; 91% being free of AF at 1 year and 84% of these after only 1 procedure in a mixed cohort of patients with paroxysmal AF (n=57) and persistent AF (n=64)[9]. Unfortunately these results have not been replicated elsewhere and, as stated above, a recent meta-analysis found no benefit of CFAE ablation in paroxysmal AF but increased freedom from atrial arrhythmias when CFAE were ablated in addition to pulmonary vein isolation in patients with non paroxysmal AF (62% vs 47%, p=0.02) [25].

## WHERE ARE WE NOW?

**Fig. (4):**
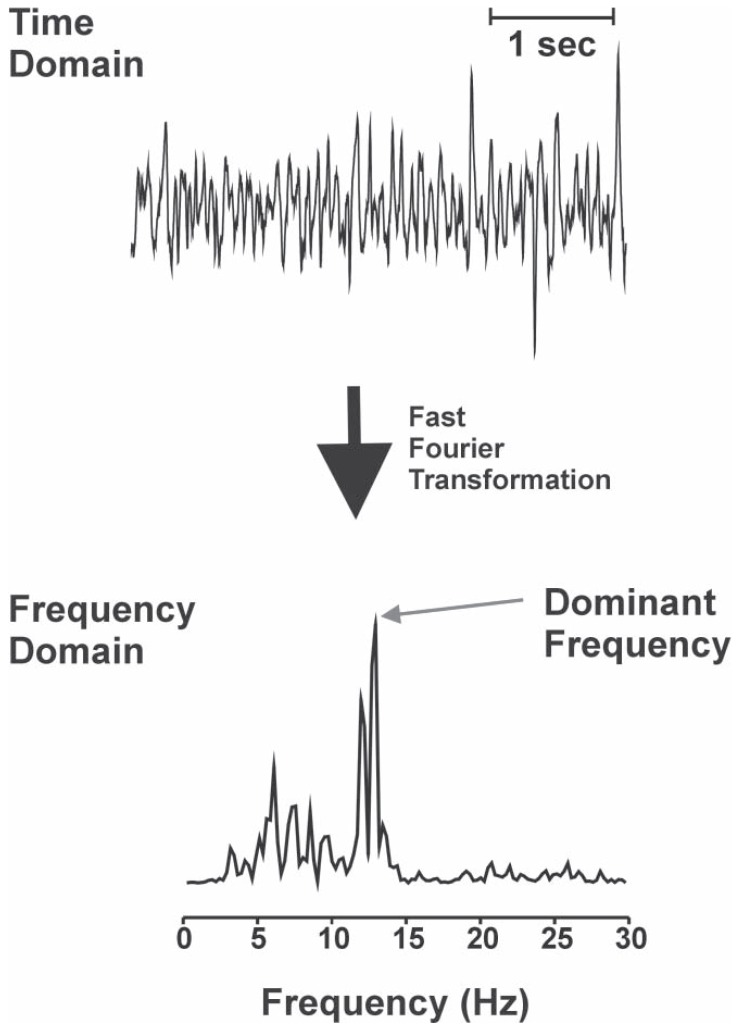
Diagram of time vs. frequency domain analysis. The top digital signal is transformed into the frequency domain (bottom panel) using Fast Fourier Transform. From this the peak or dominant frequency can be easily appreciated.

Whilst ablation of CFAE certainly has a role in non-paroxysmal AF [25] the challenge we are now facing is how to distinguish the CFAE from healthy bystander tissue (that we assume is not pathogenic) from that in diseased substrate tissue thought to be responsible for AF perpetuation. Two possible ways to analyse the recorded digital signals of AF are in the time domain and frequency domain. The time domain is how we “normally” appreciate and interpret signals i.e. how the amplitude of a signal changes with time. In contrast, the frequency domain subdivides the signal up into the individual frequency components irrespective of time (Fig. **4**). Such a plot is known as a power spectrum with the abundance of each frequency being called its power. Converting a signal into the frequency domain generally assumes sine wave periodicity. Clinical work performed with dominant frequency has had mixed results which likely stems from the problems of employing the assumptions of periodicity to aperiodic signals [30]. Calculated frequency maps do not correlate with observed signal and thus operators must rely on the map rather than the ‘live’ signals recorded prior to ablation. For these reasons the more intuitive time domain assessment has been adopted in most studies with the attempts to differentiate CFAE complexity.

Recently Hunter *et al. *demonstrated that whilst ablating some complex signals resulted in slowing of the atrial activation others would have no effect at all [17]. This elegant study first ranked the CFAE according to complexity and then ablated according to the level of complexity in either a “top down” or “bottom up” fashion; that is to say they either targeted the most complex signal first and then progressed to the more simplified signals, or they ablated the simplest signal first before targeting the increasingly complex signals. No matter which approach the AF only slowed when the most complex signals were targeted. This not only suggests that we should be more targeted in our CFAE ablation but also supports the notion that CFAE ablation is not just debulking of the atrial tissue [17].

Others have tried to differentiate substrate by looking at temporal stability of the fractionation. Whilst CFAE are known to be relatively stable over a long period of time [31] there is still an element of variability. Both Sahgy *et al*. [32] and Jadidi [33] *et al. *investigated the idea of fractionation of electrograms recorded during sinus rhythm helping to identify substrate targets and both concluded that they were not viable targets. Jadidi *et al. *assessed the fractionation in sinus rhythm (SR), during coronary sinus (CS) pacing and in AF in 9 patients with paroxysmal AF and 9 with persistent AF [33]. They found very little commonality in terms of location of fractionation between the three maps in each patient (3-25%). Interestingly, they observed that in the non-AF recordings, the fractionation occurred at collision points that were dependent on the direction of atrial activation. For instance in CS pacing the fractionation was mostly on the roof, whereas in SR it was mostly under the LIPV. In a similar vane, Saghy *et al. *compared the LA fractionation in SR to AF in 20 persistent AF patients and referenced this to the left atrial SR fractionation found in 9 non AF patients undergoing left sided ablation (4 concealed pathways, 3 WPW and 1 atrial tachycardia)[32]. Again they found little overlap between fractionation in SR and AF with a non significant correlation factor = 0.2. They found most SR fractionation to be in the LA septum or anterior wall and found no significant difference in the patterns between control and AF patients. However this is perhaps not surprising given the cycle lengths observed in AF are considerably shorter than during SR or pacing as the coupling interval of impulses are important in the pathogenesis of complex electrograms [34].

So fractionation in non-AF conditions does not help to identify substrate targets, but could the morphological stability of the fractionation during AF guide us? This concept was investigated by Ciaccio *et al. *in 10 patients with paroxysmal AF and 10 patients with persistent AF [35]. To do this, electrograms during AF were recorded for 10s in 6 standardised locations as guided by CARTO and ultrasound (4x antral outside PVs, 1x mid posterior wall, 1x anterior ridge just below left atrial appendage). In this study the regularity of the CFAE was assessed in the frequency domain by 2 novel indices (i) the regularity index - an index which compared the power of the peak frequency to the overall power in the spectral analysis and (ii) the organisational index - which evaluated the power in the peak frequency and its harmonics compared to the power in the full spectra. The repetitiveness was then assessed both in the time and frequency domains. Temporal assessment was performed using linear prediction, where template mapping of segments of the signal was performed throughout the signal to look for the degree of matching within the signal. In the frequency domain the repetitiveness was assessed by Fourier reconstruction. Here the CFAE is first transformed into its frequency components, then the frequency components are selected in order of strength, and then transformed back into the time domain. In this study, the 300 strongest frequencies were selected and transformed back into a time based signal. The repetitiveness was assessed by how similar the original signal was to the reconstructed one. With all these calculations the authors showed that CFAE signals were more uniform and repetitive across the LA in persistent than paroxysmal AF. By comparison in paroxysmal AF there was a low degree of repeatability and more randomness suggesting a more bystander role. Unfortunately there was no targeted ablation based on this analysis so the usefulness of targeting more repetitive CFAE is unknown.

Could medical alteration of the CFAE help to differentiate substrate from bystander? Working on this hypothesis, Singh *et al. *gave low dose ibutilide to 11 persistent AF patients in an attempt to *“organise areas of passive activation and not affect areas critical to AF maintenance, thereby potentially minimising the ablation lesion set”*[36]*.* In this study, the pulmonary veins were first isolated (all patients remained in AF at this stage) then CFAE maps were created with EnSite both pre and post IV ibutilide (0.25-1mg). 3 patients converted from AF to atrial tachycardia (AT) or flutter with ibutilide which were mapped and ablated. In the 8 patients still in AF, ablation was then targeted to the second, post ibutilide CFAE regions until SR was achieved. In these 8 patients the surface area of CFAE reduced with ibutilide (49-72% dependent on location). Four of the 8 patients transformed into a macroreentrant AT which was mapped and ablated, 3 required additional ablation in the right atrium and converted to SR, whilst the final patient remained in AF and had to be electrically cardioverted. Three patients required a repeat procedure due to recurrent AF/AT but after median follow-up of 455 days 72% were free from AF, a result similar to that observed with more extensive ablation lesions sets. The drawback of this study was the lack of a control group, an issue that will be resolved in the current MAGIC AF study, an international, multicentre prospectively randomised study that aims to recruit 100 patients in each arm to more fully assess the utility of ibutilide in this manner [37].

## FRACTIONATION ABLATION IN THE FUTURE?

Today the strategies to ablate persistent AF differ from centre to centre, and even from operator to operator within centres. Most ablators will start with complete antral PV isolation, but if the patient remains in AF what happens next is as heterogeneous as the CFAE themselves. Diagnostic maps performed after ablation may be inaccurate due to oedema from ablation lesions and lessen the efficacy of a guided CFAE ablation approach. Importantly much work needs to be done to correctly identify and target the CFAE associated with perpetuation of AF and CFAE resulting from ‘normal’ wavefront collision in ‘bystander’ tissue passively activated during AF. The most promising measure to date is the identification of signals that possess more continuous activity [17,18] but as yet such measures have not been employed in automated mapping systems. Another possibility is identification of reduced voltage of the tissue, but a number of studies have delivered contrasting results [18,33,38,39] and again the definition of CFAE and the population studied are likely contributing factors in the lack of consensus. To guide our practice we need more research into how to differentiate substrate from bystander tissue. This may involve abandoning CFAE detection as we currently apply it and moving into more frequency based detection; however this would require the use of contemporary techniques that may require more computational time. 

## CONCLUSION

Catheter ablation of persistent/permanent AF is growing in popularity and use of linear lesions or CFAE ablation is generally accepted as being necessary. The evidence today suggest that signal complexity reflects a complex milieu of normal tissue response to short cycle lengths and sites critical to maintenance of atrial fibrillation. The use of automated CFAE algorithms in mapping systems has resulted in the adoption of CFAE site ablation in many centres. It is important the operators understand the limitations of these systems and the putative mechanisms for CFAE in AF. While catheter ablation of CFAE improves success we have much to learn in regards of targeting these sites to reduce complications, procedure time and recurrence. We are in need of guidelines to guide mapping strategies, definitions of CFAE and ablation outcomes. Certainly some form of substrate targeted ablation is here to stay.

## Figures and Tables

**Table 1.** Co-location of Common CFAE and Parasympathetic Ganglia [24]

Location of CFAE	Proximal Coronary Sinus	Superior Vena Cava SVC– RA Junction	Septal Wall to Right PVs	Postero-superior Wall Medial to Left PVs
Parasympathetic ganglia	Posteromedial LA ganglion	Superior RA ganglion	Posterior RA ganglion	Superior LA ganglion
